# Targeting of surface alpha-enolase inhibits the invasiveness of pancreatic cancer cells

**DOI:** 10.18632/oncotarget.3572

**Published:** 2015-03-14

**Authors:** Moitza Principe, Patrizia Ceruti, Neng-Yao Shih, Michelle S. Chattaragada, Simona Rolla, Laura Conti, Marco Bestagno, Lorena Zentilin, Sheng-Hui Yang, Paola Migliorini, Paola Cappello, Oscar Burrone, Francesco Novelli

**Affiliations:** ^1^ Center for Experimental Research and Medical Studies (CeRMS), Azienda Universitaria Ospedaliera Città della Salute e della Scienza di Torino, Turin, Italy; ^2^ Department of Molecular Biotechnology and Health Sciences, University of Turin, Turin, Italy; ^3^ National Institute of Cancer Research, National Health Research Institutes, Tainan City, Taiwan; ^4^ Molecular Biotechnology Center (MBC), University of Turin, Turin, Italy; ^5^ International Centre for Genetic Engineering and Biotechnology (ICGEB), Trieste, Italy; ^6^ College of Medical Science and Technology, Taipei Medical University, Taipei City, Taiwan; ^7^ Department of Clinical and Experimental Medicine, University of Pisa, Italy

**Keywords:** pancreatic cancer, ENO1, plasminogen, monoclonal antibody, adeno-associated virus

## Abstract

Pancreatic Ductal Adenocarcinoma (PDAC) is a highly aggressive malignancy characterized by rapid progression, invasiveness and resistance to treatment. We have previously demonstrated that most PDAC patients have circulating antibodies against the glycolytic enzyme alpha-enolase (ENO1), which correlates with a better response to therapy and survival. ENO1 is a metabolic enzyme, also expressed on the cell surface where it acts as a plasminogen receptor. ENO1 play a crucial role in cell invasion and metastasis by promoting plasminogen activation into plasmin, a serine-protease involved in extracellular matrix degradation. The aim of this study was to investigate the role of ENO1 in PDAC cell invasion. We observed that ENO1 was expressed on the cell surface of most PDAC cell lines. Mouse anti-human ENO1 monoclonal antibodies inhibited plasminogen-dependent invasion of human PDAC cells, and their metastatic spreading in immunosuppressed mice was inhibited. Notably, a single administration of Adeno-Associated Virus (AAV)-expressing cDNA coding for 72/1 anti-ENO1 mAb reduced the number of lung metastases in immunosuppressed mice injected with PDAC cells. Overall, these data indicate that ENO1 is involved in PDAC cell invasion, and that administration of an anti-ENO1 mAb can be exploited as a novel therapeutic option to increase the survival of metastatic PDAC patients.

## INTRODUCTION

Pancreatic Ductal Adenocarcinoma (PDAC) is the fourth leading cause of cancer mortality in developed countries. Despite the available treatment, PDAC has the worst prognosis of all major malignancies, with a 5-year survival rate of 6% and a median survival of 6 months after diagnosis [1, 2]. The high mortality rate associated with PDAC is almost equal to the incidence rate, and is caused by the high frequency of metastatic disease found at diagnosis [3, 4].

The plasminogen system is involved in tumor growth, invasion and metastasis [5-7]. Urokinase (uPA) and tissue (tPA) plasminogen activators released from cancer cells catalyze the proteolytic conversion of plasminogen to plasmin, leading to degradation of the extracellular matrix (ECM), thus facilitating cancer cell invasion [5-8]. The uPA receptor (uPAR) is a cell membrane-anchored protein which aids the accumulation of plasminogen at the cell surface [5]. Binding proteins for plasminogen include alpha-Enolase (ENO1), Annexin 2 (ANX2) and Cytokeratin 8 (CK8) [9]. Among these, ENO1 has been classified as a pancreatic cancer-associated antigen as it is overexpressed in PDAC and induces both humoral and T cell-specific responses in patients [10, 11].

In this study, a multiple approach was adopted to investigate the role of ENO1 in the invasion and metastasis of PDAC, and to develop possible therapeutic options, based on ENO1 regulation, aimed to counteracting the invasiveness of this tumor. We evaluated i) the expression of ENO1, uPA and uPAR and of plasminogen-induced migration, in a panel of eight PDAC cell lines; ii) the *in vitro* and *in vivo* effects of anti-ENO1 monoclonal antibodies (mAbs); iii) the *in vitro* and *in vivo* effects of ENO1 silencing or mutations of its plasminogen-binding site, and iv) the effect of administering recombinant adeno-associated viral vector (AAVV) for the expression of complete anti-ENO1 mAb in *in vivo* metastatization.

## RESULTS

### Analysis of ENO1, uPAR and uPA expression in PDAC cell lines

**Figure 1:**
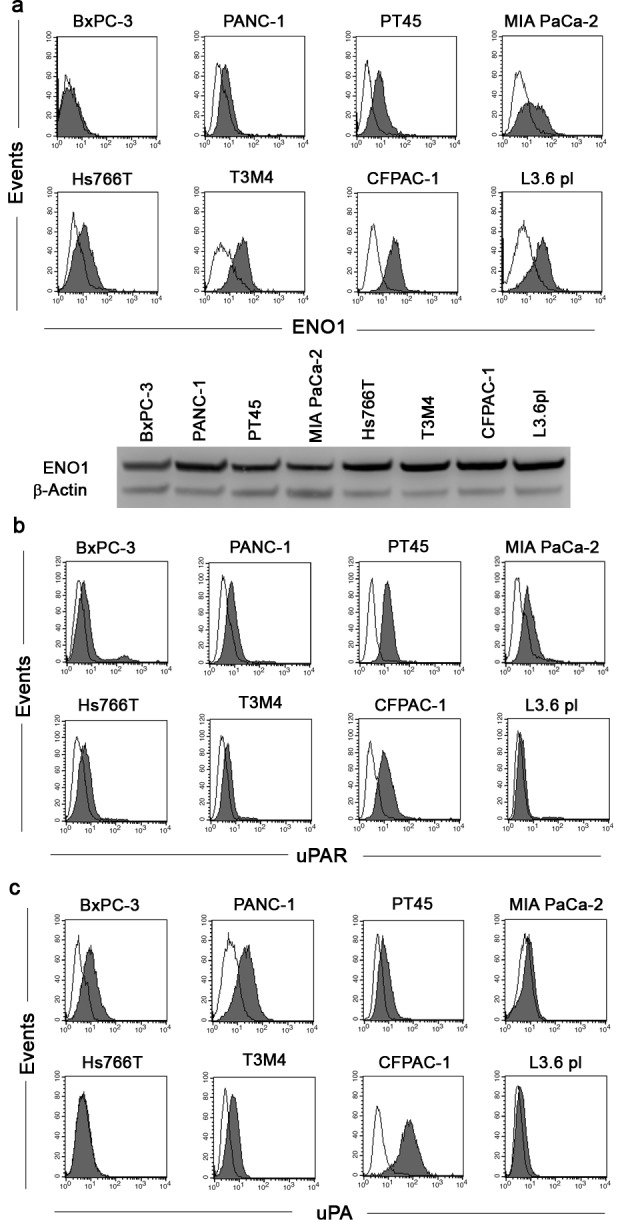
Analysis of ENO1, uPAR and uPA expression in PDAC cell lines PDAC cell lines were incubated with anti-ENO1 72/1 mAb (solid histogram a), anti-uPAR antibody (solid histogram b), anti-uPA (solid histogram c) or an isotype-matched control antibody (open histogram) and analyzed by flow-cytometry. To evaluate intracellular expression of ENO1 (a, lower panel), Western blot analysis was performed on whole cell lysates of all PDAC cell lines with anti-ENO1 72/1 mAb. Results were normalized using β-Actin. A representative of three independent experiments is shown.

Flow-cytometry, using specific 72/1 mAb, revealed that ENO1 was expressed on the surface of the majority of the tumor cell lines tested, namely PT45, MIA PaCa-2, Hs766T, T3M4, CFPAC-1, and L3.6pl. High ENO1 expression was found in T3M4, CFPAC-1 and L3.6pl, cells; there was intermediate ENO1 expression in MIA PaCa-2, Hs766T, and PT45 cells, and low or no ENO1 expression in BxPC-3 and PANC-1 cells (Fig. 1a upper panel). By contrast, all cell lines expressed similar levels of total ENO1 (Fig. 1a lower panel).

In addition to plasminogen receptors, such as ENO1, plasminogen activation requires the plasminogen activation system, as such, uPA and uPAR expression in PDAC cell lines was evaluated. After flow-cytometry analysis, we observed high levels of uPAR in PT45 and CFPAC-1 cells, intermediate levels in BxPC-3, PANC-1, MIA PaCa-2, and Hs766T cells, and low or zero levels in T3M4 and L3.6pl cells (Fig. 1b). uPA expression was high in BxPC-3, PANC-1 and CFPAC-1 cells, intermediate in PT45, and T3M4 cells, and low or absent in MIA PaCa-2, Hs766T and L3.6pl cells (Fig. 1c).

### Effect of the blockade of ENO1 on plasminogen-dependent invasion of PDAC cells

In the presence of plasminogen, CFPAC-1 cells were strongly invasive compared to those in the absence of plasminogen (Fig. S1a and b). No increase in invasion was observed in the presence of plasminogen for any of the other cell lines (Fig. S1a, b). As the CFPAC-1 cells produced uPA and expressed both surface uPAR and ENO1, they were able to invade in response to plasminogen. Nevertheless, as TGF-β has been shown to up-regulate both uPA and uPAR [12], its effect on plasminogen-dependent invasion was evaluated. In ENO-1 expressing T3M4 and in L3.6pl cells, TGF-β increased the expression of uPAR and uPA (Fig. S1c) and rendered them responsive to plasminogen-dependent invasion (Fig. S1d and Table S1).

**Figure 2:**
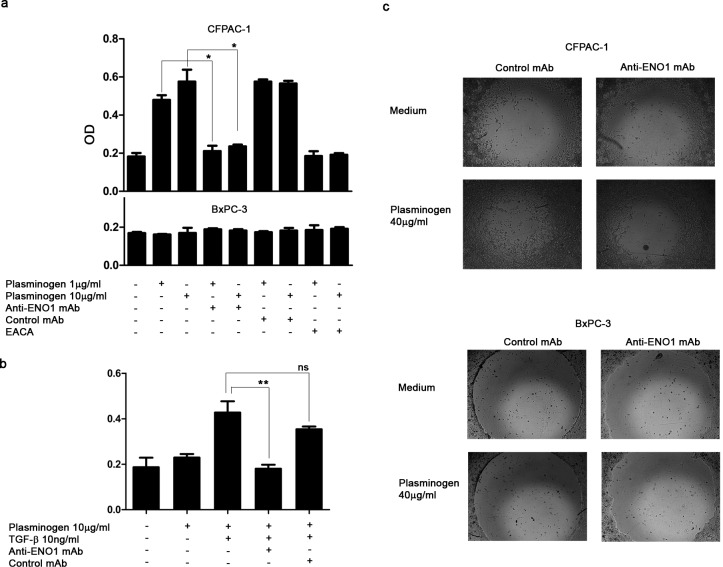
Anti-ENO1 72/1 mAb inhibits plasminogen-dependent invasion of PDAC cells (a) CFPAC-1 (upper panel), BxPC3 (lower panel) and T3M4 (b) were placed on Matrigel-coated transwell filters and plasminogen (1 μg/ml or 10 μg/ml), anti-ENO1 mAb 72/1 (50 μg/ml) or an isotype-matched IgG1 mAb (50 μg/ml), EACA (50mM) and TGF-β (10 ng/ml) were added in appropriate conditions. Data are reported as mean ± SEM of Optical Density units (OD) and the different conditions were repeated in triplicate. (c) Effect of anti-ENO1 72/1 mAb on migration in Matrigel (Oris^TM^ Platipus kit) of CFPAC-1 (upper panel) and BxPC3 (lower panel) cultured in the presence of 50 μg/ml of anti-ENO1 or IgG1 control mAb with or without plasminogen (40 μg/ml). Images are taken at x5 magnification. A representative of three independent experiments is shown. *p<0.05; **p<0.01;***p<0.001.

In the presence of anti-ENO1 mAb, the plasminogen-dependent invasiveness of both CFPAC-1 (Fig. 2a) and TGF-β-treated-T3M4 (Fig. 2b) cells was significantly reduced. The extent of this reduction was similar to that induced in CFPAC-1 cells by the plasminogen system inhibitor EACA (Fig. 2a). By contrast, BxPC-3 cells, which expressed very low levels of ENO1, did not invade in the presence of plasminogen, and were not affected by the addition of anti-ENO1 mAb (Fig. 2a lower panel). These results were also confirmed using the Oris ^TM^-FLEX Platypus Kit, in which cells were completely plunged into Matrigel and their invasion was evaluated in the absence of chemotactic stimuli, by measuring their ability to fill a central hole in the well (Fig. 2c).

In the presence of plasminogen, a similar growth pattern was observed when PDAC cells were cultured with anti-ENO1 mAb or isotype-control Ab (Fig. S2). This ruled out the possibility that the inhibitory effect of the anti-ENO1 mAb on migration is due to interference with the growth of tumor cells.

### Effect of mutation of ENO1 plasminogen binding sites on the plasminogen-dependent invasion of PDAC cells

ENO1 expression was silenced in CFPAC-1 cells with a lentivirus delivering an shRNA targeting ENO1 or the 3′UTR (shENO1). A scrambled shRNA (shCTRL) was used as a control. Both ENO1 mRNA (Fig. S3a upper panel) and protein levels (Fig. S3a lower panel), as well as plasminogen-induced invasion (Fig. S3b) were efficiently reduced after silencing in CFPAC-1 shENO1 cells. Infection of CFPAC-1 cells with a second shRNA targeting the ENO1 CDS region (shENO1#2) gave similar results (Fig. S3). All subsequent experiments were carried out using CFPAC-1 shENO1 cells.

FACS analysis revealed that uPA and uPAR expression was not modified by ENO1 silencing in CFPAC-1 cells (Fig. S3c).

**Figure 3:**
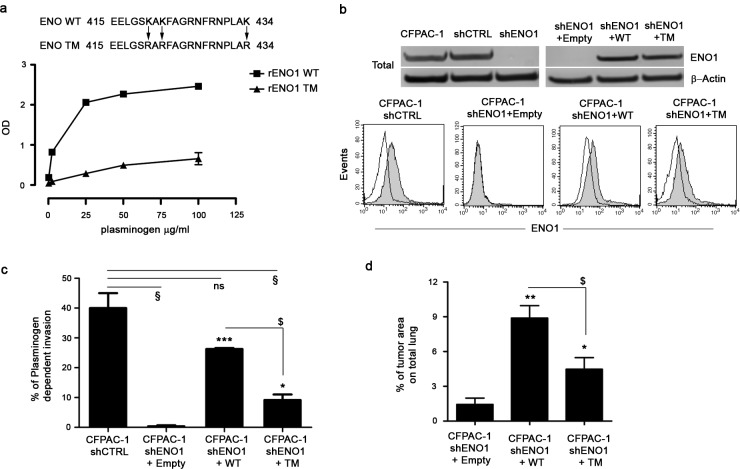
Plasminogen-dependent invasion of CFPAC-1 shENO1 transfected with WT, TM or Empty vector (a) Upper panel, a schematic representation of plasminogen-binding sites on ENO1 bearing mutations on lysines 420, 422 and 434 is shown. Lower panel, plasminogen binding to recombinant ENO1 WT or rENO1 TM was evaluated by ELISA. Data are reported as mean ± SEM of Optical Density units (OD) and the different conditions were repeated in triplicate. (b) CFPAC-1 shCTRL, CFPAC-1 shENO1+Empty, shENO1+WT, shENO1+TM cell lines were checked for the expression of ENO1 in the total cell lysates (b, upper panel). Results were normalized using β-Actin. For determining surface expression, cells were incubated with anti-ENO1 72/1 mAb (solid histogram) or an isotype-matched control antibody (open histogram) and analyzed by flow-cytometry (b, lower panel). (c) Invasion of CFPAC-1 shCTRL, shENO1+WT, shENO1+TM or shENO1+Empty cells in the presence or absence of 10 μg/ml plasminogen. Results represent the percentage of plasminogen-dependent invasion calculated as: (OD of migrated cells in the presence of plasminogen / OD of migrated cells in the absence of plasminogen) x100. (d) Growth of CFPAC-1 shENO1+WT, shENO1+TM and shENO1+Empty cells in the lungs of i.v. injected NSG mice. Bars represent the percentage of tumor area calculated as: (tumor area / total area) x100. Data are reported as mean ± SEM of five mice per group. *p<0.05; **p<0.01;***p<0.001 Statistic analysis respect to CFPAC-1 shENO1+Empty (*), to CFPAC-1 shENO1+WT ($) or to CFPAC-1 shCTRL (§).

To assess the contribution of ENO1 to PDAC invasion, shENO1 cells were transfected with a mutated form of ENO1 (shENO1+TM), in which three lysines of the plasminogen binding site at the C-terminal [13] were substituted with three arginines, resulting in a non-functional plasminogen binding site (Fig. 3a). shENO1 cells transfected with a wild type full-length exogenous ENO1 (shENO1+WT) or Empty vector (shENO1+Empty) were used as a controls. WB analysis showed that shENO1+WT or shENO1+TM rescued ENO1 protein levels (Fig. 3b upper panel). Flow cytometric analysis showed a lack of ENO1 surface expression in shENO1 and shENO1+Empty (Fig. 3b lower panel and not shown) whereas ENO1 surface expression in shENO1+WT or shENO1+TM was rescued (Fig. 3b lower panel). These data demonstrated that the triple mutation in the plasminogen binding site resulted in the inability of ENO1 to bind plasminogen without its cell surface expression being affected.

Cells were then tested for invasive capacity in response to plasminogen. Control shENO1 CFPAC-1 cells failed to invade through the Matrigel, whereas shENO1+WT cells recovered this ability, but to a lesser extent compared to CFPAC-1 shCTRL cells (Fig. 3c). Notably, shENO1+TM cells significantly reduced invasion in response to plasminogen compared to the shENO1+WT cells (Fig. 3c). To confirm the contribution of ENO1 to *in vivo* invasion and metastasis, NSG immunocompromised mice were injected i.v. with shENO1+TM, shENO1+WT or shENO1+Empty CFPAC-1 cells. On day 28, post-mortem observations confirmed a significantly reduced metastatic area in the lungs of mice injected with shENO1+Empty or with shENO1+TM cells versus mice injected with shENO1+WT CFPAC-1 cells (Fig. 3d).

### Anti-ENO1 mAb blocks liver metastasis in an orthotopic pancreatic tumor model

**Figure 4:**
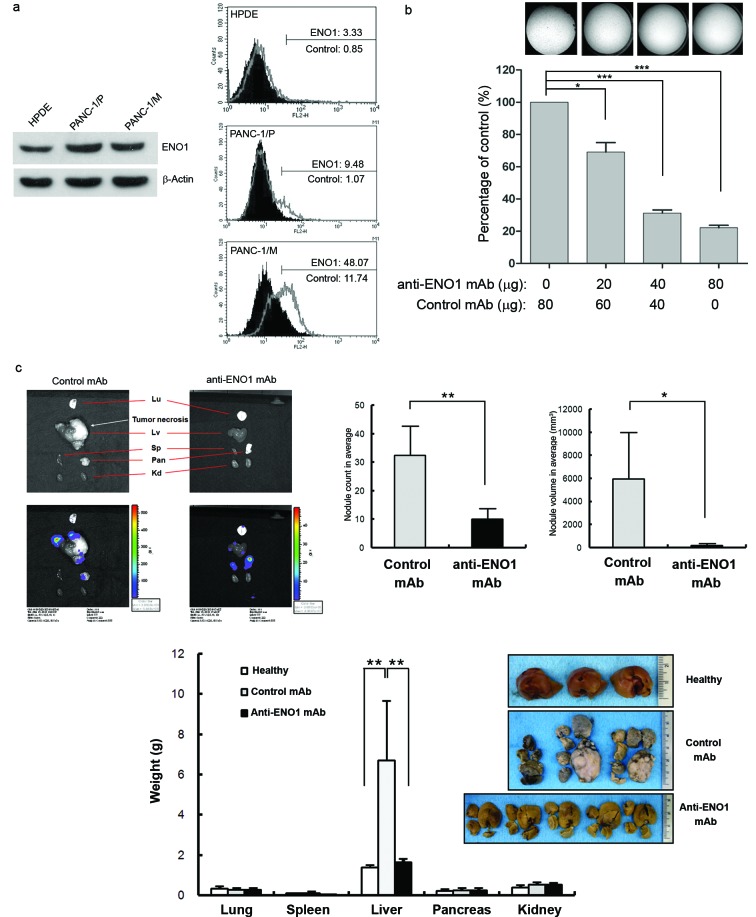
Capacity of the anti-ENO1 E10A mAb to inhibit *in vitro* and *in vivo* invasion of pancreatic cancer cells (a) Left panel, Western blot analysis of total ENO1 expression in HPDE, PANC-1/P and PANC-1/M cells using an in-house purified rabbit antiserum against ENO1. β-Actin was used as a loading control. Right panel, flow-cytometric analysis of cell-surface ENO1 in HPDE, PANC-1/P and PANC-1/M cells using anti ENO1 E10A (empty area) or its isotype-control Ab (black area). (b) Dose-dependent inhibition of cell invasion by the anti ENO1 E10A mAb in PANC-1/M cells. Cells that degraded tumor-associated matrix and migrated through the membrane were stained and quantified with the ImageJ image-processing software. One representative pair is shown in the upper panel. Data are expressed as means ± SEM and are represented as a fold-decrease in the invasive ability of cells treated with different doses of the anti ENO1 E10A mAb, compared with that of cells treated with the control antibody (lower panel). (c) Blockade of liver metastasis by the anti-ENO1 E10A mAb in an orthotopic pancreatic tumor model. Intravenous administration of the anti-ENO1 E10A (250 μg/mouse) or control mAbs was performed at 2 h and 24 h after tumor inoculation. The tissue distribution of the luciferase-expressing cells was monitored using the IVIS image system every 2 weeks for a total of 6 weeks. Mice were sacrificed after 6 weeks. Organs from one pair of representative mice treated with the anti-ENO1 or control mAbs, as indicated, were photographed (upper left). Metastatic tumors in different organs were visualized by exposure for 10s and 1s, respectively, in the luminescent mode of the IVIS system. Upper right; after mice were sacrificed, the number and volume of metastatic tumor nodules in livers of mice treated with the anti- ENO1 (*black*) or control (*gray*) mAbs were quantified. Data are expressed as the mean ± SEM of each group of mice. Lower panel, intact individual organs obtained from mice treated with the anti-ENO1 (*black*) or control (*gray*) mAbs were weighed, as indicated at the bottom. Quantitative data from each treatment are presented in the histograms. Normal livers taken from age-matched, untreated mice (*white*) served as healthy controls; * and ** indicate *P*<0.05 and *P*<0.01, respectively. For the *in vivo* experiment, five mice per group were used.

To better characterize the role of ENO1 in *in vivo* tumor spreading, PANC-1/P cells with low expression of surface ENO1 were orthotopically injected into pancreases of NOD-SCID mice. Metastatic cells were harvested from livers and cultured, and were designated as PANC-1/M. Western blot analysis of total ENO1 protein showed that its expression level in PANC-1/P cells was significantly up-regulated, compared to that in normal-like human pancreatic duct epithelial cells, HPDE (Fig. 4a left). Conversely, although the total amount of ENO1 was not increased in metastatic PANC-1/M cells, its surface distribution was clearly augmented in these cells (Fig. 4a right), confirming that surface ENO1 may play a crucial role in tumor metastasis.

To confirm the potential therapeutic use of an anti-ENO1 Ab for metastatic PDAC, experiments were conducted with a different anti-ENO1 mAb, namely E10A. The invasion of metastatic PANC-1/M cells treated with the anti-ENO1 mAb was significantly suppressed in a dose-dependent manner (Fig. 4b). In addition, these cells were orthotopically injected into the pancreases of NOD-SCID mice, followed by i.v. administration of the anti-ENO1 mAb or its isotype mAb at 2h and 24h post-inoculation. Metastatic tumor cells were fluorescently tracked by the IVIS system, showing that, in the orthotopic model, the liver was the major target for tumor metastasis of PANC-1/M cells, although a few metastatic colonies were detected in lungs and spleens (Fig. 4c upper left). Although two control mice died at 2 weeks prior to the completion of the experiments, the number and tumor volume of visible metastatic colonies in livers of mice treated with the anti-ENO1mAb were markedly decreased compared to mice treated with the control mAb (Fig. 4c upper right). This observation was further confirmed by directly weighing each organ. Again, treatment with the anti-ENO1 mAb substantially reduced metastatic tumor masses in livers (Fig. 4c lower panel). The average weights of the livers of the anti-ENO1 mAb-treated mice were comparable to those of age-matched un-injected mice.

### Anti-ENO1 mAb reduces the *in vivo* growth and metastasis of CFPAC-1 cells

**Figure 5:**
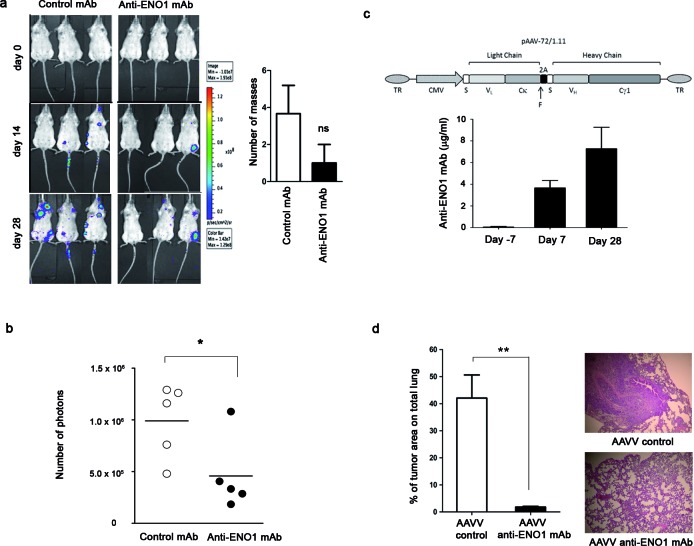
Effect of multiple injection or single injection of AAVV-anti-ENO1 72/1 mAb in the pancreatic mouse model Effect of biweekly i.p. injections of anti-ENO1 72/1mAb or an IgG1 isotype-matched control mAb (500 μg per mouse) in SCID-beige (a) or NSG (b) mice injected i.v. with luciferase-expressing CFPAC-1 cells were monitored by *in vivo* imaging. Image shows three representative mice for each treatment group followed during the experiment (day 0, day 14 and day 28). Graph shows the mean ± SEM number of tumor masses from each out of five mice. (b) From day 0 to day 14, tumor growth was monitored with IVIS spectrum technology. Results are expressed as absolute number of emitted photons at day 14. (c) Upper panel, schematic representation of the recombinant AAV vector expressing the anti-enolase 72/1.11 IgG1. TR: AAV terminal repeats sequences; CMV: cytomegalovirus promoter; S: secretion signal; V_L_: mAb 72/1.11 V_L_ region; Cκ: mouse κ constant region; F: furin cleavage site; 2A: FMDV peptide 2A sequence; V_H_: mAb 72/1.11 V_H_ region; Cγ1: mouse γ1 constant region. Lower panel, evaluation of anti-ENO1 mAb concentration in sera from NSG mice intramuscularly injected with AAVV-anti-ENO1 72/1 mAb and after 7 days i.v. with CFPAC-1 by ELISA. (d) On day 28 after tumor challenge, mice were sacrificed and lung metastases were stained with hematoxylin-eosin and counted. Bars represent the percentage of tumor area calculated as follows: (tumor area / total area) x 100. Representative images of lung sections from control and treated mice are shown. For the *in vivo* experiment, five mice per group were used. *p<0.05; **p<0.01;***p<0.001.

To prove the therapeutic effect of anti-ENO1 mAb, SCID-beige mice were injected i.v. with luciferase-expressing CFPAC-1 cells, and treated biweekly until sacrifice with anti-ENO1 mAb or isotype-matched control mAb. Notably, CFPAC-1 cells resulted in large masses at the lymph node level, prior to lung tumors. Anti-ENO1 mAb treatment led to a reduced number of tumor masses compared to control treatment. This effect was most evident from day 14 onwards, and the difference between anti-ENO1 mAb-treated mice and control mice was even greater on day 28 (Fig. 5a left). Post-mortem observations confirmed a reduced number of tumor masses in anti-ENO1 mAb-treated mice compared to control mice (Fig. 5a right). Only a few mice developed lung metastasis (4 out of 15 mice), confirmed by hematoxylin-eosin stained lung sections (data not shown). This number of mice was too small to appreciate significant differences in the number and size of lung metastasis between treated and control groups.

An additional experiment was performed using NSG mice. Mice were pre-treated for 3 days with anti-ENO1 or control Abs prior to tumor challenge. At day 0, NSG mice were injected i.v. with luciferase-expressing CFPAC-1 cells, and treated biweekly with the mAbs until sacrifice. As early as day 14 after tumor injection, anti-ENO1 mAb-treated mice emitted a significantly reduced number of photons compared to the control group, as evaluated by IVIS spectrum technology (Fig. 5b).

### An AAVV strongly increases the anti-tumor role of anti-ENO1 mAb

To further enhance the effect of anti-ENO1 mAb by continuous production of the antibody, NSG mice were injected with 1×10^11^ genocopies of Adeno-Associated Viral (AAV) vector expressing anti-ENO1 72/1 mAb, or control AAV, into femoral muscle, 7 days prior to i.v. CFPAC-1 cell injection (Fig. 5c upper panel). At day -7, 7 and 28, blood from mice was taken and analyzed for the presence of anti-ENO1 mAb. A progressively increasing concentration of anti-ENO1 mAb was observed, showing that AAVV facilitated a continuous, long-lasting and sustained production of circulating anti-ENO1 mAb (Fig. 5c lower panel). On day 28, mice injected with AAVV expressing anti-ENO1 mAb showed a significant decrease in lung metastases compared to control mice (Fig. 5d).

## DISCUSSION

Evidence from experimental models suggests that cell-associated plasminogen and its activators play a central role in tumor invasion [5, 14-16]. Numerous extracellular proteins have been identified as plasminogen receptors, including ENO1, ANX2 and CK8 [9], which are often de-regulated in cancer.

In this study, we identified ENO1 on the surface of human PDAC cells. Notably, among the eight cell lines tested, ENO1 was expressed at intermediate or high levels in metastatic cell lines (Hs766T [17], T3M4 [18], CFPAC-1 [19], L3.6pl [20]), and was absent or expressed at lower levels in primary tumor-derived cell lines (BxPC3 [21], PANC-1 [22], PT45 [23], Mia-PaCa2 [24]). *Ex vivo* analysis of PANC-1 cells from a liver metastasis showed that the surface expression of ENO1 was higher compared to the parental cells from the primary tumor. This suggests that spreading and invasion of PDAC cells is strictly related to the high cell surface expression of ENO1, which, in turn, facilitates binding of elevated concentrations of plasminogen at the cell surface. Plasminogen expressed at the cell surface activates plasmin, increasing the ability of PDAC cells to degrade the ECM. However, the mechanism by which ENO1 is expressed at the cell surface is still unknown. Hypoxia, a condition that characterizes tumor growth *in vivo*, up-regulates ENO1 expression [25-27]; therefore, we cannot rule out that the surface expression of ENO1 results from a general increase of ENO1 transcription and translation. However, as ENO1 is phosphorylated, methylated and acetylated [26, 28] in PDAC cells, the role of these post-translational modifications in the regulation of surface localization of ENO1 in PDAC cells should also be considered.

In this study, we demonstrated that the *in vitro* and *in vivo* blockade of ENO1 by treatment with two different specific mAbs reduced the migration and invasion capacity of PDAC cells. Indeed, the transduction of wild type ENO1, but not ENO1 carrying the mutated plasminogen-binding site, restored the plasminogen-dependent invasion of CFPAC-1 cells that had been previously suppressed due to ENO1 silencing. Taken together, these data strongly support the notion that ENO1 is involved in plasminogen-dependent invasion of PDAC.

*In vitro*, CFPAC-1 cells displayed an invasive ability in the presence of plasminogen, which could be ascribed to the endogenous expression of uPA and uPAR, as well as ENO1. Moreover, after exposure to TGF-β ENO1-expressing T3M4 and L3.6pl cells were induced to up-regulate uPA and uPAR and to invade in response to plasminogen (Table S1). As the invasion of all these cell lines was inhibited by anti-ENO1 mAb this implies that ENO1 regulated the PDAC metastatic process *in vivo* where the TGF-β is provided [12, 29-33]. Metastatic PANC-1/M cells derived from a liver metastasis, following orthotopical injection of PANC-1 cells in the pancreas, expressed higher levels of surface ENO1 compared to the primary tumor cells, suggesting a role for surface ENO1 in facilitating tumor spreading.

*In vivo*, blockade of ENO1 by two specific anti-ENO1 mAbs reduced tumor spreading in different mouse xenograft tumor models. In the SCID-beige tumor model, we observed a particular pattern of tumor dissemination, as cells grew in lymph-nodes without forming organ metastasis. Since PDAC cells express CXCR4, they can migrate towards the gradient of CXCL12 released by lymphoid organs and localize in lymph nodes [34]. By contrast, NGS null mice injected with CFPAC-1 cells did not develop lymph-node masses, probably because they lack functional lymph-nodes, but displayed classical lung tumor spreading. In both cases, specific treatment with anti-ENO1 mAb was effective in inhibiting tumor growth. Finally, in NOD-SCID mice that were orthotopically injected with PANC-1/M cells, the anti-ENO1 mAb was effective in inhibiting the spreading of liver metastasis.

The innovative use of AAV technology to increase the efficacy of anti-ENO1 mAb treatment in mice is noteworthy, and results in dramatic inhibition of metastasis in the NSG model. AAVV is non-pathogenic and the recombinant vector retains none of the viral genes, making it a safer alternative to live bacterial strains. The lack of the Rep gene of the AAV vector also limits its integration potential, and the vast majority of AAV vectors are thought to remain episomal [35]. This strategy has many advantages, namely resistance to the effects of pH; a localized or broad cellular tropism depending on the AAVV serotype; efficient gene transfer; persistence of gene expression, and low toxicity *in vivo*. Moreover, AAV-based therapeutic strategies have been tested in humans, and several clinical trials have been shown to be successful in terms of initial safety and proof of concept [36].

Increased expression of ENO1 has been observed in many tumors [9, 26, 37, 38], together with its ability to induce an immune response both *in vitro* and *in vivo* [10, 39-42]. Recently, we have demonstrated that ENO1 is expressed on the surface of lung tumor cells and promotes ECM degradation and invasion through a plasminogen-dependent process [16]. Our findings strongly suggest that surface ENO1 is involved in the invasion of PDAC cells, and that blockade of the ENO1/plasminogen interaction, by using an AAVV-ENO1 mAb, could provide a new therapeutic approach for the treatment of metastatic PDAC patients.

## MATERIALS AND METHODS

### Cell culture

Human PDAC cell lines used: CFPAC-1, MIA PaCa-2, BxPC-3 (all from ECACC), T3M4, PANC-1, Hs766T, L3.6pl and PT45 (all kindly provided by Prof. Paola Nisticò, Regina Elena National Cancer Institute, Rome, Italy). Cell lines were cultured in DMEM (Lonza, Milan, Italy) supplemented with 10% FBS (Lonza), L-Glutamine (GE Healthcare, Milan, Italy) and 50μg/ml of gentamicin (Gentalyn 40mg/ml, Essex Italia, Segrate, MI, Italy) at 37°C in a 5% CO_2_ atmosphere. Cells were detached using a solution of PBS containing 0.25% Trypsin-EDTA. HPDE cells were cultured in Keratinocyte-SFM medium (Invitrogen, Carlsbad, CA) supplemented with pituitary extract and recombinant EGF. PANC-1/P cells were generated by transduction of PANC-1 cells with dual reporter lentiviral particles (GeneCopoeia, Rockville, MD) to co-express GFP and firefly luciferase (Luc), followed by selection of stable transfectants using BD FACSJazz (BD Biosciences, San Jose, CA). PANC-1/M cells were obtained from metastatic liver tumors developed in an orthotopic pancreatic mouse model using PANC-1/P cells.

### Flow cytometric analysis

Cells (1×10^5^) were incubated with a primary mAb or an isotype-matched negative control antibody for 20 min at 4°C. The following primary Abs were used: mouse anti-human ENO1 72/1 IgG1 mAb (10μg/ml) [43] and rabbit anti-human uPAR polyclonal antibody (10μg/ml; Santa Cruz Biotechnology by D.B.A. Italia, Segrate, MI, Italy). An irrelevant murine IgG1 was used as a negative control (Dako, Milan, Italy). Cells were then incubated, accordingly, with a secondary biotinylated rabbit polyclonal anti-mouse F(ab')2 Ig antibody (20 min at 4°C) followed by phycoerythrin (PE)-conjugated streptavidin (SAPE), or Alexa Fluor 488 Goat anti-Rabbit IgG (H+L) antibody (LifeTechnologies, Monza, MB, Italy), for 20 min at 4°C. For detection of uPA, cells were fixed with 4% paraformaldehyde (20 min at room temperature (RT) and then permeabilized with 5% saponin. Cells were incubated with a mouse anti-human uPA mAb (0.5 μg/ml; abcam by Prodotti Gianni, Milan, Italy). An irrelevant murine IgG1 was used as a negative control (Dako). Cells were then incubated with a secondary PE goat anti-mouse IgG antibody (BioLegend by Campoverde, Milan, Italy) for 20 min at 4°C. Following this, cells were re-suspended in Dulbecco's Phosphate-Buffered Saline (DPBS), acquired with a FACSCalibur and analyzed using the CellQuest program (BD Bioscience, Buccinasco, MI, Italy). PANC-1/P, PANC-1/M and HPDE cells were stained with the mouse anti-human ENO1 E10A IgG2a mAb or its isotype control (1.5 μg/mL) for 30 min on ice, visualized with goat anti-mouse F(ab^'^)_2_ conjugated PE (2.5 μg/mL; Jackson ImmunoResearch, Cambridgeshire, UK), and analyzed using the FACScan flow cytometer (BD Biosciences).

### Western blot analysis

PDAC cells (1×10^7^) from the various cell lines were harvested, lysed, resolved and transferred to nitrocellulose membranes, as previously described [40]. Membranes were incubated for 1 h at RT with anti-ENO1 72/1 mAb or rabbit polyclonal anti- β-Actin antibody (Sigma-Aldrich), at dilutions of 1:2000 in Tween-Tris-Buffered Saline (TTBS) and then probed with a horseradish peroxide (HRP)-conjugated anti-mouse IgG (Santa Cruz) or HRP-conjugated goat anti-rabbit Ig secondary antibody (Sigma-Aldrich) at dilutions of 1:2000. For Western blot analysis of ENO1 in PANC-1/P, PANC-1/M and HPDE cells, membranes were probed with in-house purified rabbit antiserum against ENO1 or with mouse antibody specific to β-Actin (Sigma St. Louis, MO, USA) as a protein loading control. Immunocomplexes were detected by probing with appropriate secondary antibodies conjugated with HRP (Jackson ImmunoResearch), and were visualized using the SuperSignal detection system (Thermo Fisher, Waltham, MA, USA).

### *In vitro* chemo-invasion assay

Porous (8 μm pore size) tissue culture inserts (6.5 mm diameter) (Costar by Sigma-Aldrich) were coated with 50μl of Matrigel (BD Biosciences) diluted at 1:8 in serum-free DMEM, and incubated for 4 h at 37°C. Inserts were placed in a 24-well plate containing 0.6 ml/well of DMEM supplemented with 3% FBS. Cells (1×10^5^/well) were seeded in triplicate in serum-free medium in the upper chamber and incubated for 48h at 37°C. Plasminogen Glu-Type (1μg/ml or 10μg/ml; Calbiochem by D.B.A.), anti-ENO1 72/1 mAb or IgG1 isotype-matched control antibody (50μg/ml; anti-intracellular domain of the IFN-γR2, γR37) [44], Epsilon-Amino-Caproic Acid (EACA) (50mM; Calbiochem) and TGF-β (1ng/ml or 10ng/ml; Peprotech by Tebu-bio, Magenta, MI, Italy) were added to appropriate inserts. After incubation, invasive cells were fixed in 2% glutaraldehyde, stained with Crystal Violet (Sigma-Aldrich), treated with a 10% acetic acid solution (Sigma-Aldrich), and eluates were read at 570nm with a spectrophotometer (Bio-Rad laboratories, Segrate, MI, Italy). For analysis of the HPDE, PANC-1/P and PANC-1/M cell lines, 5×10^4^ cells/well were seeded and incubated, as described above, with anti-ENO1 E10A or with control mAb. After incubation for 24 h, cells were fixed, stained with Gimsa, and quantified by the ImageJ analysis [45]. Invasion assays in the presence of plasminogen alone (40μg/ml) or with anti-ENO1 72/1 mAb (50μg/ml) or with mouse IgG1 control (50μg/ml) were also carried out with a Cell Migration Assembly Kit, Oris^TM^ - FLEX (Platipus Technology by TEMA Ricerca, Bologna, Italy) according to the manufacturer's instructions.

### Silencing of ENO1 in the PDAC cell line CFPAC-1

Two Mission short hairpin RNA (shRNA), one targeting the 3′UTR of the gene coding for ENO1 (TRCN0000029324) and one targeting the CDS region (TRCN0000029327) were used to transform bacteria (Sigma-Aldrich, Milan, Italy); plasmids were purified with the PureLink HiPure Plasmid Maxiprep Kit (LifeTechnologies). Lentiviruses were produced by co-transfecting 293T packaging cells (Clontech by Diatech Lab Line Srl, Jesi, AN, Italy) with pLKO.1 puro vector containing the shRNA and the helper vectors pCMVΔ8.74 (Add gene, Cambridge, MA, USA) and pVSV-G (Clontech), using the calcium phosphate method. Lentiviruses collected at 24h after transfection were used for the transduction of the CFPAC-1 cell line supplemented with 8 μg/ml polybrene (Sigma-Aldrich) and, after 48h of infection, cells were then selected for stable silencing using 2 μg/ml Puromycin (Sigma-Aldrich). For quantitative mRNA expression analysis, a polymerase chain reaction (PCR) was carried out with total cDNA and the SYBR Green PCR Master Mix (LifeTechnologies), with a two-step amplification protocol. mRNA expression of target genes was normalized using the mRNA level of β-Actin.

### Plasmid construction and mutagenesis

The total RNA from CFPAC-1 cell lines was extracted using the RNeasy Mini kit (Qiagen, Milan, Italy). RNA concentration and purity was determined using a NanoDrop instrument (Thermo Scientific by VWR, Milan, MI, Italy), and 1μg of the total RNA was used as a template for cDNA synthesis using the iScript cDNA synthesis kit (BioRad, Segrate, MI, Italy). Using the specific primers (Table S2) encoding for ENO1, cDNA was amplified by PCR; amplification products were analyzed on 1% agarose gels, and isolated from gels by using a Gel Extraction Kit (Qiagen), ligated with the EGFP retroviral vector Pallino-GFP [46], using the XhoI and NotI restriction sites, transformed into Top10 competent cells (LifeTechnologies), and sequenced. A mutated form of ENO1, bearing mutations in its plasminogen binding sites on lysines, 420, 422 and 434 (substituted by arginines) was obtained by three different point mutations using primers containing K420R, K422R and K434R substitutions (Table S2) and the QuikChange site-directed mutagenesis kit (Stratagene by Eppendorf, Milan, Italy); real-time PCR was performed using the C 1000 thermal cycler (BioRad). The PCR product was subsequently subjected to sequencing.

### Establishment of the PDAC cell line CFPAC-1 expressing ENO1 and the ENO1 mutant variant

The retrovirus was obtained by transfecting the Pallino-GFP vector containing the ENO1 gene or the ENO1 mutant variant (K420R, K422R and K434R) into the GP-293 packaging cells (Clontech) co-transfected with the pVSV-G helper vector (Clontech) using the calcium phosphate method. Released retroviruses were collected at 24h after transfection, and used for transduction of the 3′UTR ENO1-silenced CFPAC-1 (shENO1) cell line in the presence of 8 μg/ml polybrene (Sigma-Aldrich). After 12h of incubation, complete medium was added and cells were cultured for a further 2 days. Cells were then analyzed for GFP content on a FACS Calibur flow cytometer (BD Biosciences). The CELLQuest TM software (BD Biosciences) was used for data acquisition and analysis of the cells expressing the full-length exogenous ENO1 (shENO1+WT), the triple-mutated ENO1 (shENO1+TM), as well as cells expressing the empty vector (shENO1+Empty) as a control. Expression of protein levels was also analyzed by Western blotting.

### Enzyme-Linked Immunosorbent Assay (ELISA)

The plasminogen-binding assay was performed with a recombinant human ENO1 (rENO1) protein histidine-tag (rENO1 WT) or a mutated form (rENO1 TM), produced as previously described [10]. Briefly, rENO1 (2.5 μg/mL in 0.1 M Na_2_CO_3_) was coated in 96-plate well and incubated over-night at 4°C. After 2 h of blocking with PBS 3% bovine serum albumin (BSA) at RT, plasminogen diluted in PBS 1% BSA 0.05% Tween was added at different doses for 1 h at RT. After incubation with Streptavidin-HRP (Sigma) and then with tetramethylbenzidine (TMB) (Sigma), the plate was read at the spectrophotometer at a wavelength of 450nm.

Anti-ENO1 mAb levels were measured by ELISA, by binding to rENO1 (2 μg/mL in 0.1 M Na_2_CO_3_). Sera collected before the injection of AAVV and 2 and 5 weeks after the injection were diluted at 1:100 in DPBS, and antibody concentrations were calculated by regression analysis using seven 2-fold serial dilutions starting from 1μg/mL of anti-ENO1 72/1 mAb for creating a standard curve [39].

### Construction of the recombinant adeno-associated viral vector (AAVV) for the expression of complete anti-ENO1 72/1 mAb

Total RNA was extracted from hybridoma 72/1 cells [43] using the RNeasy Mini Kit (Qiagen), and V_L_ and V_H_ genes were amplified by RT-PCR with primer pairs LB13 (GAYATTGTGATGACYCAGKC) / Cκ2 (TGGATACAGTTGGTGCAGC) and VHB (AGGTSMARCTGCAGSAGTCWGG) / CHγ (GGCCAGTGGATAGAC), respectively. Amplified fragments were sequenced and re-amplified with primer pairs VL12-BssHII (ATAGCGCGCCGTTTCAGCTCCAGCTTGGT) / VL12-EcoRV (ACTCGGATATCGTGATGACCCAGGCT) for V_L_ and VH14-ApaLI (TATAGTGCACTCTCAGGCCTATCTGCAGCAGT)/VH14-Eag/BspE(TAATTCCGGACGGCCGAAGAGACAGTGACCAGAGT) for V_H_, to allow the reconstitution of complete functional light- and heavy-chain genes for the anti-ENO1 mAb. Re-amplified V genes were inserted into a vector derived from pcDNA3 (Life Technologies) containing the sequences of the constant regions of the mouse κ light chain and γ1 heavy chain arranged in a single transcriptional unit, where the light chain and heavy chain genes are separated by a sequence encoding the autocatalytic peptide 2A from the FMD Virus; the two genes contained in this bicistronic mRNA are translated into a single polypeptide that spontaneously cleaves into two distinct proteins [47]. To remove the residual peptide 2A, a sequence encoding a furin cleavage site (RSKR) was introduced between the light-chain and the peptide 2A coding sequences [48]. The V_L_ sequence was inserted into BssHII/EcoRV in this bicistronic vector, in order to join it to the κ constant region gene, while the V_H_ segment was firstly inserted into ApaLI/BspEI in a pUT-SEC vector [49], in order to provide it with a sequence encoding a secretion signal; the SEC-V_H_ unit was then excised with HindIII/EagI, and joined to the mouse γ1 constant region gene in the bicistronic vector, downstream of the light chain gene and the 2A sequence. The complete anti-ENO1 mAb transcriptional unit was then transferred to HindIII/XbaI in a plasmid vector, under the control of the cytomegalovirus immediate early promoter, to yield the final vector pAAV-72/1. To confirm that this vector was able to direct the production of functional antibodies, 7 μg of pAAV-72/1 was used to transfect approximately 3×10^6^ HEK293 cells using the standard calcium phosphate method, and supernatants were used to probe cellular extracts from PDAC cells by Western blotting. The reactivity of the secreted recombinant mAb was compared to that of the original 72/1 mAb (Fig. S4). The pAAV-72/1 vector was then used for the generation of recombinant AAV (serotype9) in the AVU (AAV Vector Unit) Core Facility of the ICGEB (Trieste, Italy), as described [50].

### ***In vivo*** experiments

NOD-SCID IL2Rgamma^null^ (NSG) mice (provided by the animal facility of the Molecular Biotechnology Center, University of Turin, Italy) were injected in the tail vein (i.v.) with 1×10^5^ CFPAC-1 shCTRL or shENO1+Empty, shENO1+WT, shENO1+TM cells (in 0.1 ml DPBS) and the mice were euthanized after 28 days.

SCID-beige mice (Charles River, Calco, LC, Italy) or NSG mice were injected in the tail vein (i.v.) with 1×10^5^ CFPAC-1 cells expressing luciferase (in 0.1 ml DPBS), followed by biweekly injections of anti-ENO1 72/1 mAb (500 μg/mouse) or an isotype-matched control antibody. NSG mice were pre-treated for 3 days with the same antibodies. In a different set of experiments, NSG mice were injected 7 days before the i.v. CFPAC-1 cell challenge with 1×10^11^ genocopies of AAVV expressing anti-ENO1 72/1 mAb or control AAVV in femoral muscles. For detection of *in vivo* growth of CFPAC-1 luciferase-transduced tumor cells, mice were anesthetized with 2% isofluorane and given i.p. injections of luciferin substrate (100 mg/kg) (Caliper Life Sciences by Promega, Milan, Italy),10 min prior to imaging using the IVIS Spectrum *in vivo* Imaging System (Xenogen Corp., Alameda, CA, USA). Images were taken on day 0, 14 and 28 after CFPAC-1 injection (SCID-beige mice) and on day 0 and 14 (NSG mice). Images were analyzed using Living Image software for the IVIS Spectrum (Xenogen Corp.). After 28 days, mice were euthanized and checked for metastasis by histological analysis.

For orthotopic experiments, NOD-SCID male mice (6–8 weeks old) were obtained from the National Laboratory Animal Center, Taiwan, and housed under specific pathogen-free conditions according to the guidelines of the Animal Care Committee at the National Health Research Institutes, Taiwan. On Day 0, PANC-1/M cells expressing luciferase (1×10^6^/200 ml per mouse) were orthotopically injected into the pancreases of the mice. The mice were intravenously administered with the anti-ENO1 E10A mAb (250 μg/mouse) or its isotype control, as indicated, at 2 h and 24 h after tumor inoculation. Tissue distribution of the cells was monitored using the IVIS *in vivo* imaging system every 2 weeks for a total of 6 weeks, as described above. Tumor volumes of metastatic tumors were measured using the following formula: length (mm) × width^2^ (mm^2^) × (π/6). All organs from the mice treated with the anti-ENO1 E10A or control mAb, as well as of age-matched healthy mice were also weighed. In all experiments, five mice were used in each group.

### Tissue sample and histopathology

Mice were euthanized, necropsied and examined for the presence of tumor masses. Tumor masses and main organs, including lungs, spleens, and livers were fixed in 4% (v/v) neutral-buffered formalin (Sigma-Aldrich) overnight, transferred to 70% ethanol, followed by paraffin-embedding. For histological analysis, 5μm formalin-fixed paraffin-embedded tissue sections were cut and stained with hematoxylin-eosin. Tumor/normal tissue ratios were evaluated with ImageJ software.

### Statistical analysis

The Student's t test (GraphPad Prism 5 Software, San Diego, CA) was used to evaluate the differences in the invasion test, and in *in vivo* experiments. Values were expressed as mean ± SEM.

## SUPPLEMENTARY MATERIAL, FIGURES, TABLES


