# Supplementary material for: Targeting of surface alpha-enolase inhibits the invasiveness of pancreatic cancer cells
Source: Oncotarget. 2015 Mar 14;6(13):11098–113. doi: 10.18632/oncotarget.3572 (PMC4484442; doi:10.18632/oncotarget.3572)
Supplement: Supplementary file 1 [file oncotarget-06-11098-s001.pdf]

Targeting of surface alpha-enolase inhibits the invasiveness of pancreatic cancer cells

Supplementary Material

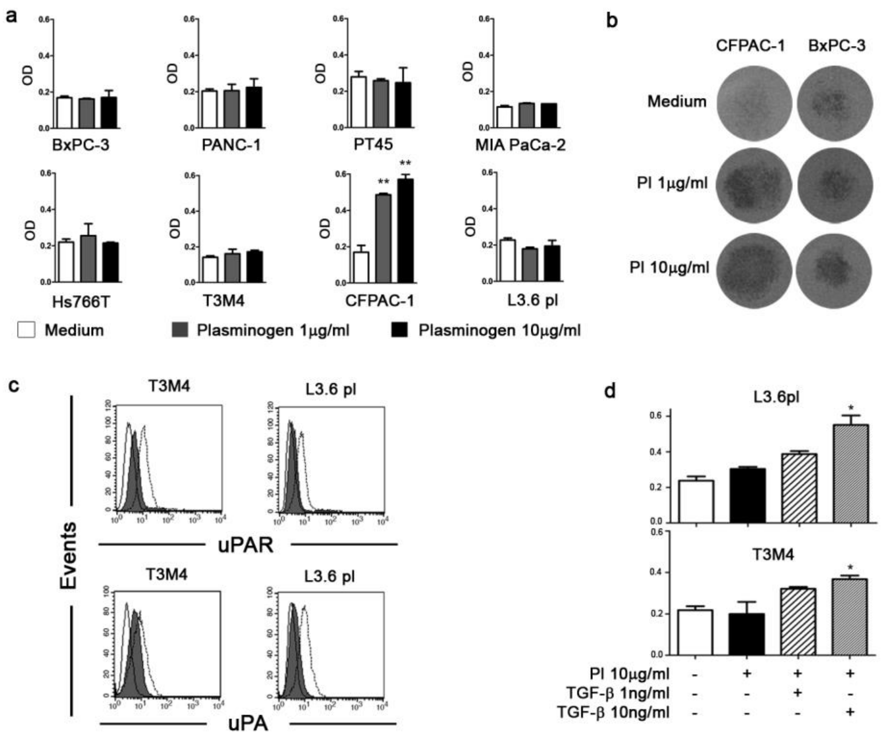

Figure. S1: Invasive potential of PDAC cell lines in presence of plasminogen. (a) PDAC cells were placed on Matrigel coated transwell filters and plasminogen added in appropriate conditions (1 μg/ml and 10 μg/ml). After 48 hours, migrated cells were fixed, stained with crystal violet and dissolved. The eluates were read at the spectrophotometer at 570 nm wavelength. Results are expressed as mean ± SEM of Optical Density units (OD) and conditions were in triplicate. CFPAC-1 invasion in response to plasminogen is significantly different compared to medium only condition. One representative of three independent experiments is shown. **(b)** A representative image of transwell membrane stained for each condition is reported for CFPAC-1 and BxPC-3 cells. **(c)** To evaluate uPA and uPAR overexpression after 24h of treatment with TGF-β (10ng/ml), flow-cytometry analysis was performed. PDAC cell line were incubated with anti-uPAR antibody for surface staining (solid histogram without TGF-β and dashed line after treatment with TGF-

β) or fixed with PFA, treated with Saponin and incubated with anti-uPA mAb (solid histogram without TGF-β and dashed line after treatment with TGF-β) for intracytoplasmic staining. Isotype matched control antibody was used as control (open histogram). One representative of three independent experiments is shown. **(d)** L3.6pl and T3M4 cells were placed on Matrigel coated transwell filters were added in appropriate conditions plasminogen (10 μg/ml) and TGF-β (1ng/ml or 10ng/ml). Experiment was analyzed as described before. PDAC cells invasion in response to TGF-β (10ng/ml) is significantly different compared to plasminogen condition. One representative of three independent experiments is shown. *$p<0.05$; **$p<0.01$;***$p<0.001$

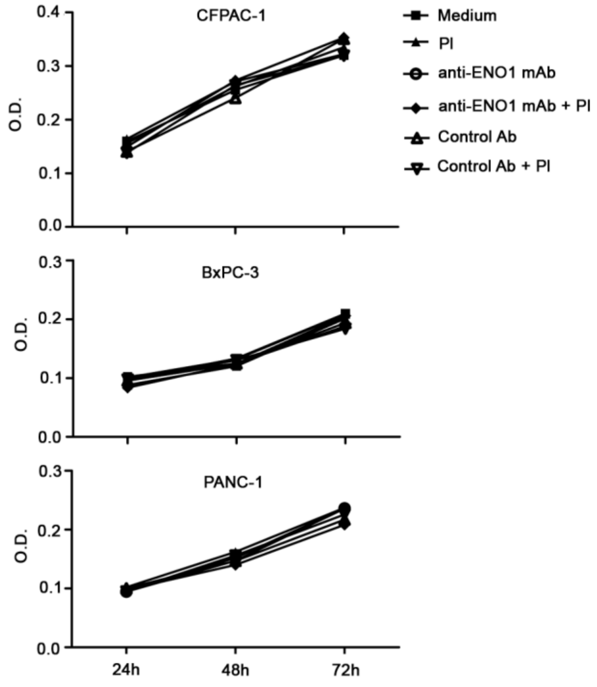

Figure. S2: Effect of anti-ENO1 mAb on proliferation. CFPAC-1, BxPC-3 and PANC-1 cells survival was assessed by MTT assay. Cells were starved and after 36h 2% FBS was added with or without plasminogen (10 µg/ml), anti-ENO1 72/1 mAb (50 µg/ml) or control Ab depending on the different conditions. MTT solution was added 24, 48 or 72 hrs after serum replenishment. O.D. values were measured at 570 nm.

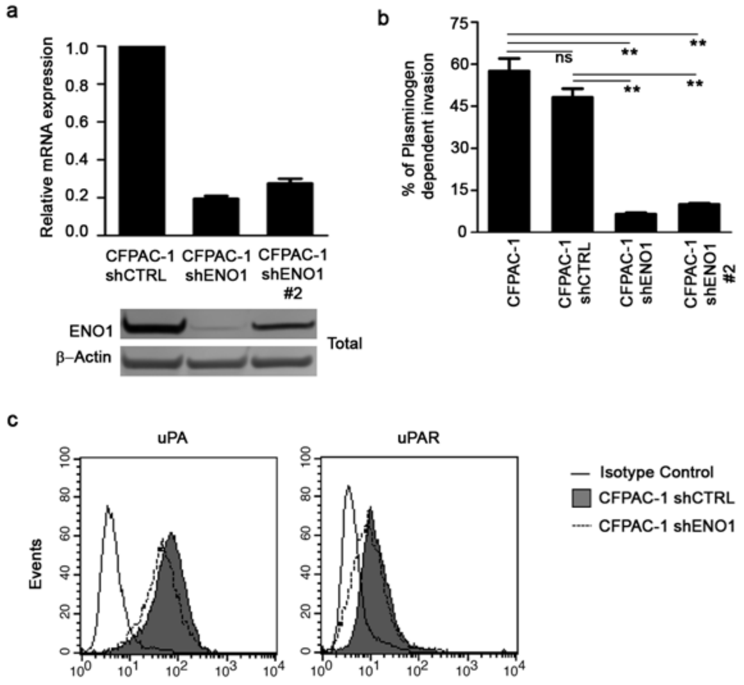

Figure. S3: Knockdown of ENO1 in CFPAC-1 cell lines. (a) To confirm the silencing of ENO1 at mRNA and protein level, Real-Time PCR **(a upper panel)** and Western Blot analysis was performed respectively **(a lower panel)**. Results were normalized using β-Actin. CFPAC-1 shCTRL cell line was used as control. **(b)** CFPAC-1 parental or shCTRL shENO1 and shENO1#2 CFPAC-1 were placed on Matrigel-coated transwell filters and plasminogen (10 µg/ml) were added in appropriate conditions. Results represent the percentage of plasminogen-dependent invasion calculated as: (OD of migrated cells in the presence of plasminogen / OD of cells migrated in the absence of plasminogen) x100. The different conditions were repeated in triplicate. **(c)** CFPAC-1 shENO1 and shCTRL cell lines were incubated with anti-uPA antibody (left panel), anti-uPAR (right panel) or isotype-matched control antibody and analyzed by flow-cytometry. One representative of three independent experiments is shown. *p<0.05; **p<0.01;***p<0.001

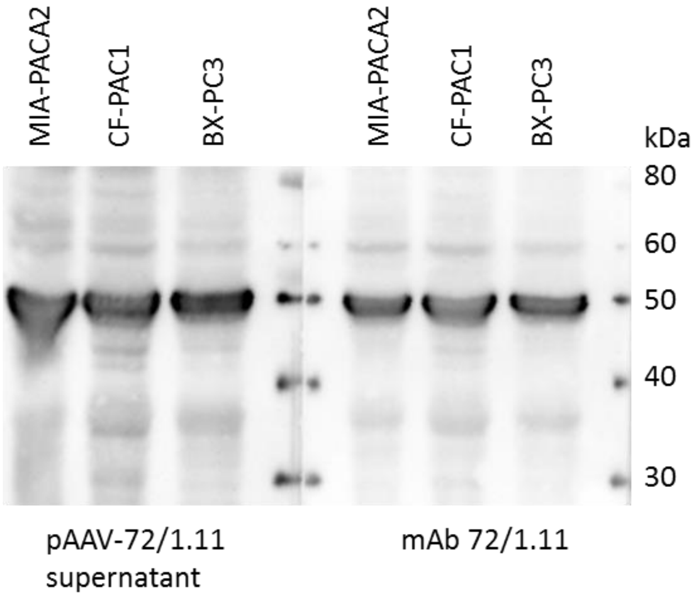

Figure. S4: Construction of the recombinant adeno-associated viral vector (AAVV) for the expression of complete anti-ENO1 mAb Western blotting of extracts from the indicated pancreatic cancer cell lines, probed with either the original mAb 72/1.11 or supernatant from HEK293 cells transfected with the pAAV-72/1.11 vector and revealed with HRP-conjugated anti-mouse IgG antibodies. The same 48 kDa band, corresponding to ENO1 is detected in all lanes.

Table S1: Plasminogen-related protein expression and invasion in PDAC cells

CELL LINE	uPA[a]	uPAR[b]	ENO1[c]	INVASION [d]
BxPC-3	+	+	-	no
PANC-1	+	+	-	no
PT45	-	+	+	no
MIA PaCa-2	-	+	+	no
Hs766T	-	+	+	no
T3M4	+	+(*)	+	Yes(*)
CFPAC-1	+	+	+	Yes
L3.6pl	+(*)	+(*)	+	Yes(*)

LEGEND

uPA [a], uPAR [b] and ENO1 [c] expression was determined by flow-cytometry as reported Fig.1. The results were arbitrarily assigned based on ΔMFI values. [d] Invasion capability was determined by invasion transwell assay as reported in Fig. S1a.

(*) after TGF-β treatment as reported in Fig. S1 c-d.

Table S2: Primers used for mutagenesis and sequencing of ENO1

Primers	*Sequence*
ENO1 XhoI forward	5'-CCGCTCGAGTATGTCTATTCTCAAGATCCA-3'
ENO1 NotI reverse	5'-TAGCGGCCGCTTACTTGGCCAAGGG
EnoK420R ,K422R K434R forward	5'-GAAGAGGAGCTGGGCAGCAGGGCTAGGTTTGCCGGCAGGAAC-3'.
Eno K420R ,K422R K434R reverse	5'-GTTCCTGCCGGCAAACCTAGCCCTGCTGCCCACGTCCTCTTC-3'.
ENO1 qPCR forward	5'-GCCTCCTGCTCAAAGTCAAC-3'
ENO1 qPCR reverse	5'-AACGATGAGACACCATGACG-3'
Beta Actin Forward	5'-CGCCGCCAGCTCASCCATG-3'
Beta Actin Reverse	5'-CACGATGGAGGGGAAGACGG-3'
SEQ 1ENO	5'-TGTACGGTGGGAGGTCTATA-3'
SEQ 2ENO	5'-GATGGATGGAACAGAAAATA-3'
SEQ 3ENO	5'-TACACTGATAAGGTGGTCAT-3'
SEQ 4ENO	5'-GTCATGGTGTCTCATCGTTC-3'
SEQ 5ENO	5'-TTCCATCCATCTCGATCATCA-3'
